# Unloading‐induced atrophy and decreased oxidative capacity of the soleus muscle in rats are reversed by pre‐ and postconditioning with mild hyperbaric oxygen

**DOI:** 10.14814/phy2.13353

**Published:** 2017-07-25

**Authors:** Ai Takemura, Roland R. Roy, Ikumi Yoshihara, Akihiko Ishihara

**Affiliations:** ^1^ Laboratory of Cell Biology and Life Science Graduate School of Human and Environmental Studies Kyoto University Kyoto Japan; ^2^ Department of Integrative Biology and Physiology and Brain Research Institute University of California Los Angeles California

**Keywords:** Forkhead box‐containing protein O1, muscle fiber size, muscle fiber type, muscle oxidative capacity, peroxisome proliferator‐activated receptor *γ* coactivator‐1*α*, unloading and reloading

## Abstract

Our aim was to determine the effects of pre‐ and/or postconditioning with mild hyperbaric oxygen (1.25 atmospheric pressure, 36% oxygen for 3 h/day) on the properties of the soleus muscle that was atrophied by hindlimb suspension‐induced unloading. Twelve groups of 8‐week‐old rats were housed under normobaric conditions (1 atmospheric pressure, 20.9% oxygen) or exposed to mild hyperbaric oxygen for 2 weeks. Ten groups then were housed under normobaric conditions for 2 weeks with their hindlimbs either unloaded via suspension or not unloaded. Six groups subsequently were either housed under normobaric conditions or exposed to mild hyperbaric oxygen for 2 weeks: the suspended groups were allowed to recover under reloaded conditions (unrestricted normal cage activity). Muscle weights, cross‐sectional areas of all fiber types, oxidative capacity (muscle succinate dehydrogenase activity and fiber succinate dehydrogenase staining intensity) decreased, and a shift of fibers from type I to type IIA and type IIC was observed after hindlimb unloading. In addition, mRNA levels of peroxisome proliferator‐activated receptor *γ* coactivator‐1*α* decreased, whereas those of forkhead box‐containing protein O1 increased after hindlimb unloading. Muscle atrophy and decreased oxidative capacity were unaffected by either pre‐ or postconditioning with mild hyperbaric oxygen. In contrast, these changes were followed by a return to nearly normal levels after 2 weeks of reloading when pre‐ and postconditioning were combined. Therefore, a combination of pre‐ and postconditioning with mild hyperbaric oxygen can be effective against the atrophy and decreased oxidative capacity of skeletal muscles associated with hindlimb unloading.

## Introduction

Hindlimb unloading in rodents results in atrophy of all fiber types, a shift of fibers from type I to type II, de novo synthesis of type IIx myosin heavy chain, reduced oxidative capacity, decreased mRNA levels of heat shock proteins and peroxisome proliferator‐activated receptor *γ* coactivator‐1*α* (*Pgc‐1α*), and increased mRNA levels of forkhead box‐containing protein O1 (*FoxO1*) in hindlimb skeletal muscles (Ishihara et al. 1997, 2004, 2008; Nagatomo et al. 2011a). These changes, however, are muscle‐ and fiber type specific, that is, predominantly slow antigravity muscles and high oxidative slow fibers are affected the greatest by hindlimb unloading. For this reason, we chose to study the soleus muscle, an ankle extensor comprised predominantly of slow type I fibers (Nagatomo et al. 2011a).

Some preconditioning factors prior to hindlimb unloading have been shown to lessen the effects of hindlimb unloading on the affected musculature in rodents. For example, a single 60‐min bout of heat stress at ~41.6°C for 6 h prior to hindlimb unloading reduces the extent of atrophy of the soleus muscle normally associated with 8 days of hindlimb unloading (Naito et al. 2000). A single 25‐min bout of treadmill running at 20 m/min and a 20° incline prior to 2 weeks of hindlimb unloading attenuates the decrease in the percentage of type I fibers in the soleus muscle although there is no effect on muscle weight (Fujino et al. 2009).

Hyperbaric oxygen therapy involves breathing 100% oxygen in a pressurized chamber, that is, a device used for medical treatment. Hyperbaric oxygen therapy is usually conducted under conditions of 2–3 atmospheric pressure with 100% oxygen. Elevation of atmospheric pressure accompanied by high oxygen concentration in the chamber enhances partial pressure of oxygen, thus increasing the amount of oxygen, especially dissolved oxygen, in blood plasma: hyperbaric oxygen therapy at 2 and 3 atmospheric pressure with 100% oxygen increases dissolved oxygen in blood plasma 14‐ and 22‐fold, respectively, compared to normobaric conditions (Tibbles and Edelsberg 1996; Leach et al. 1998). Hyperbaric oxygen therapy has been used for routine wound care, treatment of most dive injuries, and treatment of patients who are ventilated or in critical care. Nonetheless, hyperbaric oxygen therapy is associated with an increased risk of oxygen toxicity and excessive oxidative stress (Narkowicz et al. 1993; Benedetti et al. 2004; Bosco et al. 2007).

Mild hyperbaric oxygen, which is 1.25 atmospheric pressure with 36% oxygen in the chamber, does not increase oxidative stress (Ishihara et al. 2014). In addition, middle‐ear barotrauma, which is often induced by high pressure, does not occur under mild hyperbaric oxygen conditions because of the relatively low pressure and oxygen concentration in the chamber. Mild hyperbaric oxygen facilitates oxidative metabolism, particularly pathways in the mitochondrial tricarboxylic acid cycle, thus enhancing the oxidative capacity of skeletal muscles and their fibers (Ishihara et al. 2005; Matsumoto et al. 2007). Metabolic syndrome (Takemura and Ishihara 2017), lifestyle‐related diseases (Yasuda et al. 2006, 2007; Gu et al. 2010; Nagatomo et al. 2010a, 2011b), and arthritis (Nagatomo et al. 2010b) are alleviated when rodents are exposed to mild hyperbaric oxygen because of improvement of the oxidative capacity in mitochondria and consequently oxidative metabolism in tissues and organs.

Oxidative metabolism in skeletal muscle, including mitochondria biogenesis, fiber type percentage, and oxidative enzyme activity, is regulated by factors such as PGC‐1*α* (Wu et al. 1999; Miura et al. 2003; Liang and Ward 2006; Mortensen et al. 2006). Therefore, the reduced mRNA levels of *Pgc‐1α* in hindlimb unloaded skeletal muscles of rodents are related to the low percentage of high oxidative fibers and the high percentage of low oxidative fibers (Nagatomo et al. 2011a). We hypothesized that the mRNA levels of *Pgc‐1α* would be enhanced under mild hyperbaric oxygen conditions and therefore would be one of the factors that could contribute to an improvement in the decreased oxidative capacity of skeletal muscles in an unloaded state.

The purposes of the present study were to determine whether mild hyperbaric oxygen prior to and/or after hindlimb suspension‐induced unloading has pre‐ and/or postconditioning effects on (1) the recovery of the atrophy and decreased oxidative capacity in skeletal muscle associated with hindlimb unloading and (2) the mRNA levels of *Pgc‐1α* because mild hyperbaric oxygen improves oxidative metabolism in skeletal muscle, including enhanced mitochondria biogenesis and an increase in the percentage of fibers with a high oxidative enzyme activity level, which are regulated, at least in part, by PGC‐1*α*.

## Materials and Methods

### Ethical approval

All experimental and animal care procedures were conducted in accordance with the Guidelines for the Care and Use of Laboratory Animals issued by the Institutional Animal Experiment Committee of Kyoto University (Kyoto, Japan).

### Experimental animals and treatments

Eight‐week‐old male Wistar rats were subdivided into 12 groups (see Table 1 for abbreviations of conditions and groups, eight rats per group). One‐half of the rats (six groups: N, NN, NU, NNN, NUN, and NUH) were housed under normobaric conditions, that is, 1 atmospheric pressure with 20.9% oxygen for 24 h/day during 2 weeks. The other half of the rats (six groups: H, HN, HU, HNH, HUN, and HUH) were housed under normobaric conditions (1 atmospheric pressure with 20.9% oxygen) for 21 h/day and mild hyperbaric oxygen conditions, that is, 1.25 atmospheric pressure with 36% oxygen for 3 h/day (11:00–14:00 h) using a chamber with mild hyperbaric oxygen (Japan Patent No. 5076067 dated 7 September 2012; Inventor: Akihiko Ishihara) during 2 weeks.

**Table 1:** Twelve groups of male Wistar rats

Groups	Time points		
	10 weeks	12 weeks	14 weeks
N (a)	Normobaric		
H (b)	Hyperbaric		
NN (c)	Normobaric	Normobaric	
HN (d)	Hyperbaric	Normobaric	
NU (e)	Normobaric	Normobaric	
		Unloaded	
HU (f)	Hyperbaric	Normobaric	
		Unloaded	
NNN (g)	Normobaric	Normobaric	Normobaric
HNH (h)	Hyperbaric	Normobaric	Hyperbaric
NUN (i)	Normobaric	Normobaric	Normobaric
		Unloaded	Reloaded
NUH (j)	Normobaric	Normobaric	Hyperbaric
		Unloaded	Reloaded
HUN (k)	Hyperbaric	Normobaric	Normobaric
		Unloaded	Reloaded
HUH (l)	Hyperbaric	Normobaric	Hyperbaric
		Unloaded	Reloaded

Normobaric (N), 1 atmospheric pressure with 20.9% oxygen for 24 h; hyperbaric (H), 1 atmospheric pressure with 20.9% oxygen for 21 h and 1.25 atmospheric pressure with 36% oxygen for 3 h; hindlimb suspended‐induced unloading (U), which allows for free movement of the forelimbs and prevents the hindlimbs from contacting the sides or floor of the cage. Eight rats per group. Letters in parentheses are used in the Figures for group identification and to identify statistically significant differences.

Two groups (N and H: normobaric and mild hyperbaric oxygen conditions, respectively) were euthanized at the 10‐week time point. The remaining 10 groups were housed under normobaric conditions for 2 weeks with (six groups: NU, HU, NUN, NUH, HUN, and HUH) or without hindlimb suspension‐induced unloading (four groups: NN, HN, NNN, and HNH). We used the tail suspension method described by Nagatomo et al. (2011a). This method allows for free movement of the forelimbs but prevents the hindlimbs from contacting the sides or the floor of the cage. This method has been successful and none of the animals slipped out of the rigging and underwent weight bearing.

Two nonsuspended (NN and HN) and two suspended (unloaded NU and HU) groups were euthanized at the 12‐week time point. The remaining four suspended groups were reloaded (unrestricted cage activity) and allowed to recover under normobaric (NUN and HUN) or mild hyperbaric oxygen (NUH and HUH) conditions for 2 additional weeks (euthanized at the 14‐week time point). The remaining two nonsuspended groups were maintained for 2 weeks under normobaric (NNN) or mild hyperbaric oxygen (HNH) conditions.

Food and water were provided ad libitum to all the groups. The room was maintained in a controlled 12‐h light/dark cycle (dark period from 20:00 to 08:00 h) at 22°C ± 2°C and 45–55% relative humidity.

### Biochemical analyses

The soleus muscles were excised bilaterally and wet weighed. The soleus muscle of the right leg was frozen in liquid nitrogen for measurement of succinate dehydrogenase (SDH) activity as described elsewhere (Nagatomo et al. 2012b,c). Briefly, the muscle was homogenized using a glass tissue homogenizer with 5 volumes of ice‐cold 0.3 mol/L phosphate buffer, pH 7.4. The final concentrations of the components of the reaction mixture were as follows: 17 mmol/L sodium succinate, 1 mmol/L sodium cyanide, 0.4 mmol/L aluminum chloride, and 0.4 mmol/L calcium chloride. This reaction mixture was transferred to a spectrophotometer, and reduction of cytochrome *c* was quantified by monitoring the increase in extinction at 550 nm. SDH activity was calculated from the ferricytochrome *c* concentration and protein content.

### Histochemical analyses

The soleus muscle of the left leg was divided into distal and proximal portions for histochemical and mRNA analyses, respectively. The distal portion of the muscle was pinned to a corkboard near its approximate in vivo length and was rapidly frozen in isopentane precooled with a mixture of dry ice and acetone. The muscle was mounted on a specimen chuck with the Tissue‐Tek OCT compound (Sakura Finetek Japan Co., Ltd., Tokyo, Japan). Serial transverse sections (16‐*μ*m thick) were prepared on a cryostat at −25°C. The sections were brought to room temperature and preincubated under acidic (pH 4.5) or alkaline (pH 10.4) conditions for the subsequent assessment of ATPase staining intensity. The fibers in each muscle section were classified as type I (a positive response to preincubation at pH 4.5 and a negative response to preincubation at pH 10.4), type IIA (a negative response to preincubation at pH 4.5 and a positive response to preincubation at pH 10.4), or type IIC (a positive response to preincubation at pH 4.5 and 10.4) as reported elsewhere (Nagatomo et al. 2009; Takemura et al. 2016). Fiber cross‐sectional area (CSA) was measured by tracing the outline of individual fibers. The mean fiber type percentage and CSA were determined for approximately 300 adjacent fibers located in the central region of each muscle.

SDH staining intensity was quantified in the same 300 fibers in serial sections using a computer‐assisted image processing system (Neuroimaging System, Kyoto, Japan) as described elsewhere (Nagatomo et al. 2009; Takemura and Ishihara 2017). Sectional images were digitized as gray scale images. Each pixel was quantified as 1 of 256 gray levels: a gray level of 0 was equivalent to 100% light transmission, whereas a gray level of 255 was equivalent to 0% light transmission. The mean optical density of all pixels, which were converted to gray‐level values, within a fiber was determined using a calibration photographic tablet with 21 steps of gradient density ranges and the corresponding diffused density values.

### mRNA analyses

Total RNA was extracted from the proximal portion of the left soleus muscle using an extraction kit (QuickGene RNA Tissue Kit SII; Fujifilm, Tokyo, Japan). Reverse transcription was carried out by means of the High Capacity cDNA Archive Kit (Applied Biosystems, Foster City, CA), and cDNA samples were stored at −20°C. The expression levels of *Pgc‐1α* and *FoxO1* were quantified by TaqMan Gene Expression Assays (Applied Biosystems) (Nagatomo et al. 2011a). Each TaqMan probe and primer set was validated by means of a quantitative real‐time polymerase chain reaction with a series of cDNA template dilutions to obtain standard curves of threshold cycles against relative concentration using the housekeeping gene of 18S RNA as an internal standard. All the samples and nontemplate control reactions were conducted on a 7500 Fast Sequence Detection System (Applied Biosystems). The mRNA levels were normalized to those of the control group.

### Statistics

The data are expressed as mean ± standard deviation. All samples were analyzed in a blinded fashion. Student's *t* test was used to evaluate the differences between normobaric and mild hyperbaric oxygen groups at the 10‐week time point. Analysis of variance (ANOVA) was used to evaluate the differences among the four groups at the 12‐week time point and among the six groups at the 14‐week time point. When the overall differences were found to be significant by ANOVA, individual group comparisons were made by *Scheffé's* post hoc test. Statistical significance was set at *P *<* *0.05.

## Results

### Body weights

**Figure 1:**
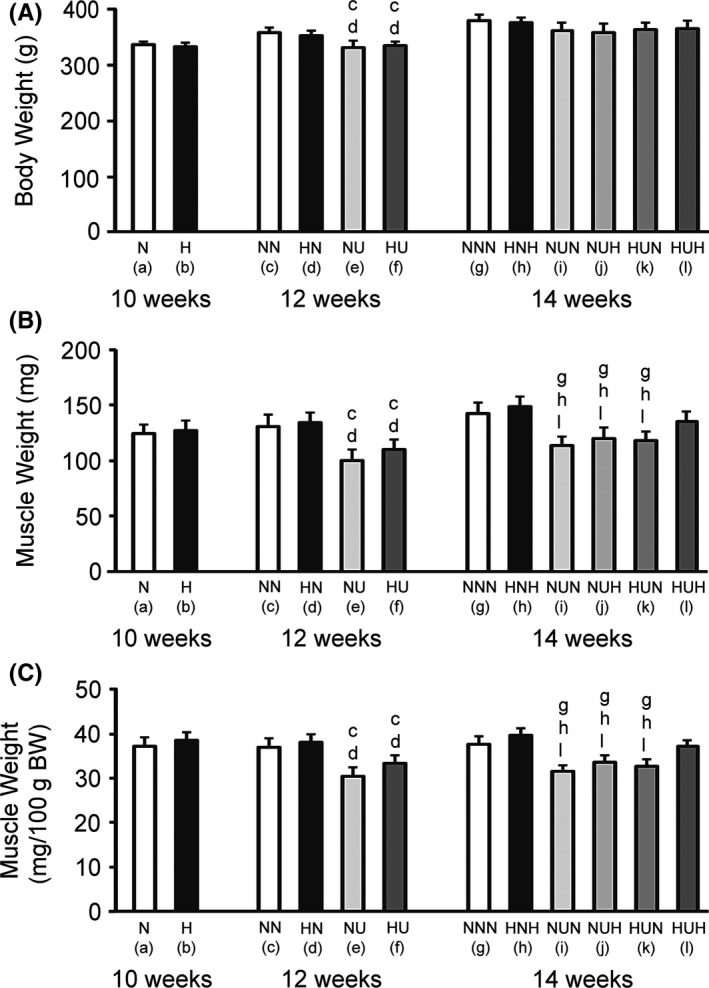
Body weights (A) and absolute (B) and relative (per body weight, C) wet weights of the soleus muscle. BW, body weight. Data are the mean and standard deviation for eight rats per group. The letters (a) to (l) below the group identifications correspond to those in Table 1. The letters above individual bars identify significant differences (*P *<* *0.05).

There were no differences in the body weight between the N and H groups at the 10‐week time point (Fig. 1A). The body weights were lower in the suspended groups (NU and HU) than in the nonsuspended groups (NN and HN) at the 12‐week time point. There were no differences in the body weight among the six groups (NNN, HNH, NUN, NUH, HUN, and HUH) at the 14‐week time point.

### Absolute and relative soleus muscle weights

There were no differences in the absolute (Fig. 1B) or relative (Fig. 1C) muscle weight between the N and H groups at the 10‐week time point. The absolute and relative muscle weights were lower in the suspended groups (NU and HU) than in the nonsuspended groups (NN and HN) at the 12‐week time point. The absolute and relative muscle weights were lower in the NUN, NUH, and HUN groups than in the NNN, HNH, and HUH groups at the 14‐week time point.

### Soleus muscle SDH activity

**Figure 2:**
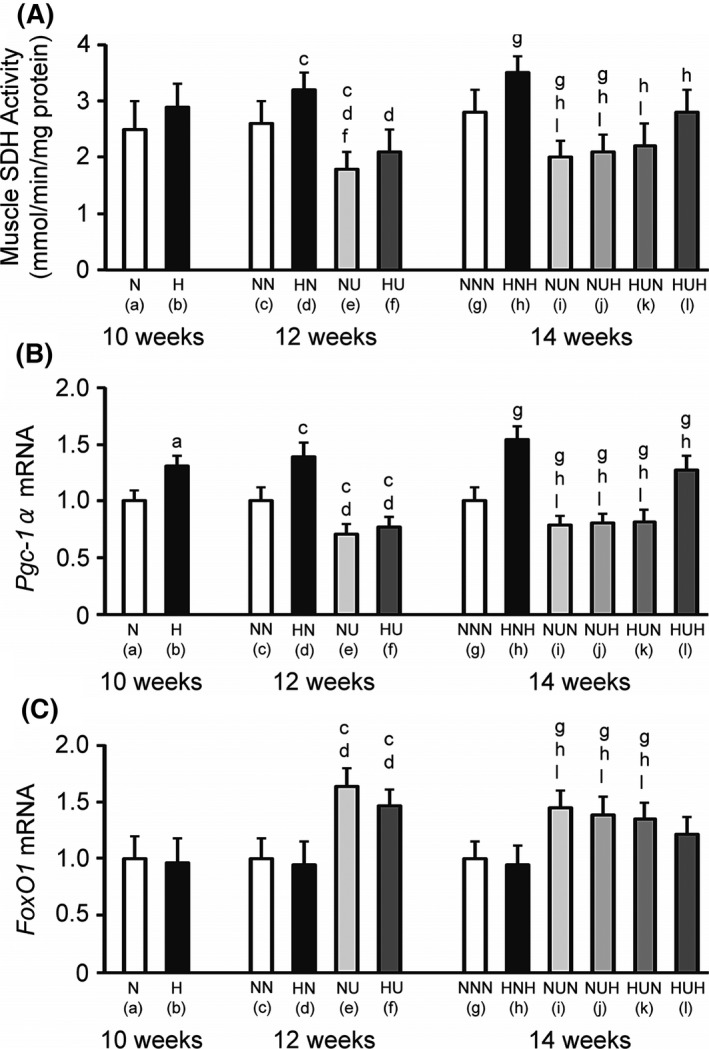
Soleus muscle succinate dehydrogenase (SDH) activity (A) and mRNA levels of peroxisome proliferator‐activated receptor *γ* coactivator‐1*α* (*Pgc‐1α*, B) and forkhead box‐containing protein O1 (*FoxO1*, C). Data are the mean and standard deviation for eight rats per group. The letters (a) to (l) below the group identifications correspond to those in Table 1. The letters above individual bars identify significant differences (*P *<* *0.05).

There were no differences in the muscle SDH activity between the N and H groups at the 10‐week time point (Fig. 2A). The muscle SDH activity was at least 0.75 mmol/(min·mg) higher in the HN group than in the other three groups (NN, NU, and HU) at the 12‐week time point. Furthermore, these values were lower in the NU group than in the NN and HU groups. The muscle SDH activity was higher in the HNH group than in the other five groups (NNN, NUN, NUH, HUN, and HUH) and higher in the HUH group than in the NUN, NUH, and HUN groups at the 14‐week time point. Furthermore, these values were lower in the NUN and NUH groups than in the NNN group.

### mRNA levels of *Pgc‐1α*

The mRNA levels *of Pgc‐1α* were higher in the H group than in the N group at the 10‐week time point (Fig. 2B). The mRNA levels of *Pgc‐1α* were higher in the HN group than in the other three groups (NN, NU, and HU) at the 12‐week time point. Furthermore, the mRNA levels of *Pgc‐1α* were lower in the NU and HU groups than in the NN group. The mRNA levels of *Pgc‐1α* were higher in the HNH group than in the other five groups (NNN, NUN, NUH, HUN, and HUH) at the 14‐week time point. The *Pgc‐1α* mRNA levels were lower in the NUN, NUH, and HUN groups than in the NNN and HUH groups. Furthermore, the mRNA levels of *Pgc‐1α* were higher in the HUH group than in the NNN group at this latest time point.

### mRNA levels of *FoxO1*

There was no difference in the mRNA levels of *FoxO1* between the N and H groups at the 10‐week time point (Fig. 2C). The mRNA levels of *FoxO1* were higher in the suspended groups (NU and HU) than in nonsuspended groups (NN and HN) at the 12‐week time point. The mRNA levels of *FoxO1* were higher in the NUN, NUH, and HUN groups than in the NNN, HNH, and HUH groups at the 14‐week time point.

### Soleus muscle fiber properties

**Figure 3:**
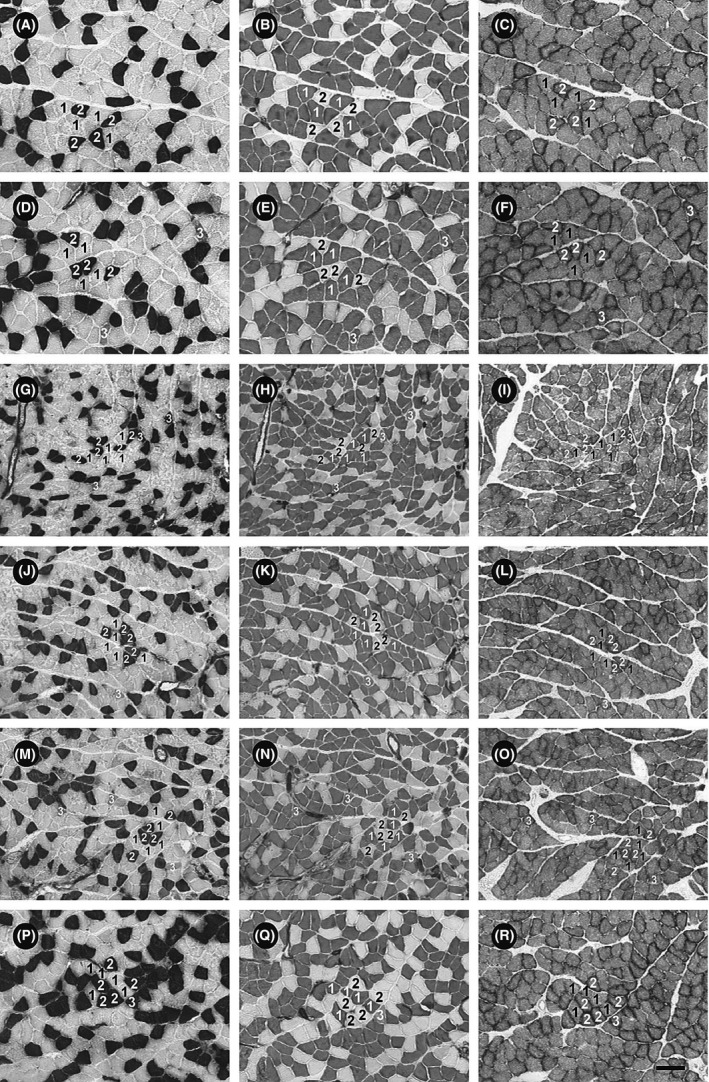
Serial transverse sections of the soleus muscles of rats from the NNN (A–C), HNH (D–F), NUN (G–I), NUH (J–L), HUN (M–O), and HUH (P–R) groups at the 14‐week time point. The serial sections were stained for adenosine triphosphatase activity after preincubation at pH 10.4 (A, D, G, J, M, and P) or pH 4.5 (B, E, H, K, N, and Q), and for succinate dehydrogenase activity (C, F, I, L, O, and R). 1 = type I; 2 = type IIA; 3 = type IIC. The scale bar in panel R is 100 *μ*m.

**Figure 4:**
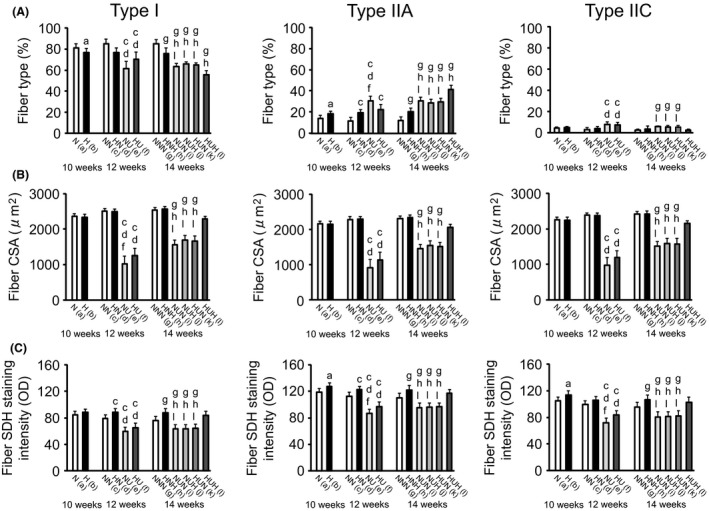
Percentages (A), cross‐sectional areas (CSAs, B), and succinate dehydrogenase (SDH) staining intensity (C) of each fiber type in the soleus muscle. OD, optical density. Data are the mean and standard deviation for eight rats per group. The letters (a) to (l) below the group identifications correspond to those in Table 1. The letters above individual bars identify significant differences (*P *<* *0.05).

Three types of fibers, that is, type I, type IIA, and type IIC, were identified in the muscles of all groups (Figs. 3 and 4A).

The percentage of type I fibers was lower and that of type IIA fibers was higher in the H group than in the N group at the 10‐week time point (Fig. 4A). The percentage of type I fibers was lower and that of type IIC fibers was higher in the suspended groups (NU and HU) than in the nonsuspended groups (NN and HN) at the 12‐week time point. Furthermore, the percentage of type IIA fibers was higher in the HN, NU, and HU groups than in the NN group and higher in the NU group than in the HN and HU groups. The percentage of type I fibers was lower and that of type IIA fibers was higher in the suspended groups (NUN, NUH, HUN, and HUH) than in the nonsuspended groups (NNN and HNH) at the 14‐week time point. Furthermore, the percentage of type I fibers was higher and that of type IIA fibers was lower in the NUN, NUH, and HUN groups than in the HUH group. The percentage of type IIC fibers was higher in the NUN, NUH, and HUN groups than in the NNN and HUH groups.

There were no differences in the CSA of any fiber types between the N and H groups at the 10‐week time point (Fig. 4B). The CSA of each fiber type was smaller in the suspended groups (NU and HU) than in the nonsuspended groups (NN and HN) at the 12‐week time point. The CSA of type I fibers was smaller in the NU group than in the HU group. The CSA of any fiber types was smaller in the NUN, NUH, and HUN groups than in the NNN, HNH, and HUH groups at the 14‐week time point.

The SDH staining intensity of type IIA and IIC fibers was higher in the H group than in the N group at the 10‐week time point (Fig. 4C). The SDH staining intensity of type I and IIA fibers was higher in the HN group than in the NN group at the 12‐week time point. The SDH staining intensity of any fiber types was lower in the suspended groups (NU and HU) than in the nonsuspended groups (NN and HN). Furthermore, the SDH staining intensity of type IIA and IIC fibers was lower in the NU group than in the HU group. The SDH staining intensity of each fiber type was higher in the HNH group than in the NNN group at the 14‐week time point. Furthermore, the SDH staining intensity of any fiber types was lower in the NUN, NUH, and HUN groups than in the NNN, HNH, and HUH groups.

## Discussion

### Effects of hindlimb unloading on the soleus muscle

The observed responses of the soleus muscle to hindlimb suspension‐induced unloading in the present study are consistent with those reported in previous studies (Ishihara et al. 1997, 2004, 2008; Nagatomo et al. 2011a). Two weeks of hindlimb unloading decreased soleus muscle weight, oxidative enzyme activity, mRNA levels of *Pgc‐1α*, and cross‐sectional areas and oxidative enzyme staining intensity of each fiber type and increased mRNA levels of *FoxO1*.

### Effects of mild hyperbaric oxygen on muscle oxidative capacity

Various studies on animal models have shown that mild hyperbaric oxygen alleviates or prevents metabolic syndrome (Takemura and Ishihara 2017), type 2 diabetes (Yasuda et al. 2006, 2007; Gu et al. 2010), diabetes‐induced cataract (Nagatomo et al. 2011b), hypertension (Nagatomo et al. 2010a), and type II collagen‐induced arthritis (Nagatomo et al. 2010b). Hyperbaric oxygen at oxygen concentrations higher than 40% has undesirable effects such as increased numbers of invasive inflammatory cells (Folz et al. 1999) and/or increased oxidative stress (Benedetti et al. 2004; Bosco et al. 2007; Nagatomo et al. 2012a). An oxygen concentration of 36%, however, does not appear to increase oxidative stress (Nagatomo et al. 2012a; Ishihara et al. 2014) and, therefore, was used for oxygenation in the present study. The observed shift toward greater amounts of oxidative fibers and the increases in fiber oxidative enzyme staining intensity and mRNA levels of *Pgc‐1α* after mild hyperbaric oxygen (the H group compared to the N group) suggest that mild hyperbaric oxygen used in the present study was effective in maintaining and improving oxidative metabolism in skeletal muscles and their fibers.

### Pre‐ and postconditioning with mild hyperbaric oxygen as a countermeasure for muscle deficits associated with hindlimb unloading

Preconditioning (HUN) or postconditioning (NUH) with mild hyperbaric oxygen alone did not lessen the effects of hindlimb unloading on the weight and properties of the soleus muscle, indicating that these interventions alone were not effective as countermeasures for observed hindlimb unloading‐induced atrophy and decreased oxidative capacity of the soleus muscle. In contrast, the combination of pre‐ and postconditioning with mild hyperbaric oxygen (HUH) attenuated the atrophy and decrease in oxidative capacity of the soleus muscle induced by hindlimb unloading. In fact, the HUH group was found to have a greater muscle weight, oxidative enzyme activity, fiber cross‐sectional areas, fiber oxidative enzyme staining intensity, and mRNA levels of *Pgc‐1α*, but lower mRNA levels of *FoxO1* as compared to all the other recovery groups (NUN, NUH, and HUN). The relatively high mRNA levels of *Pgc‐1α* in the HUH group suggest a link between mRNA levels of *Pgc‐1α* and the enhanced oxidative capacity observed in the soleus muscle during the recovery stage. Based on these results, we conclude that a combination of pre‐ and postconditioning with mild hyperbaric oxygen can be recommended for effective reversal of the atrophy and decreased oxidative capacity of skeletal muscle associated with hindlimb unloading. Since neither preconditioning nor postconditioning alone were effective countermeasures, it is likely that pre‐ and postconditioning with mild hyperbaric oxygen triggered different signaling pathways and that only a combination of these treatments activated the signaling cascades required for the recovery of soleus muscle mass and oxidative capacity.

### Safety of mild hyperbaric oxygen

Hyperbaric oxygen therapy is an established medical treatment and is usually conducted under conditions of 2–3 atmospheric pressure with 100% oxygen (Tibbles and Edelsberg 1996; Leach et al. 1998). The elevated pressure and increased oxygen concentration enhance dissolving of oxygen in blood plasma and lead to vasoconstriction and hyperoxygenation. Hyperbaric oxygen therapy is effective against a variety of medical conditions related to arterial gas embolism, acute carbon monoxide poisoning, decompression sickness suffered by divers, and thermal burns. In addition, it may be used as an adjunctive therapy for the prevention and treatment of osteoradionecrosis, clostridial myonecrosis, and compromised skin grafts and flaps (Kawamura et al. 1978; Takeshima et al. 1999; Nakada et al. 2006; Wasiak et al. 2006; Buettner and Wolkenhauer 2007).

Regardless of pressure, oxygen treatments are associated with an increased risk of oxygen toxicity and excessive oxidative stress. The latter and formation of free radicals play a key role in the pathogenesis of many diseases and complications, including atherosclerosis, cataract, retinopathy, myocardial infarction, hypertension, diabetes, renal failure, and uremia (Maier and Chan 2002; Griendling and FitzGerald 2003; Dalle‐Donne et al. 2006). Some studies on animals (Giblin et al. 1995; Padgaonkar et al. 2000) and humans (Palmquist et al. 1984; Gesell and Trott 2007) have shown that exposure to 2–3 atmospheric pressure with 100% oxygen induces myopia and cataracts. In addition, exposure to hyperbaric oxygen is believed to cause excessive production of reactive oxygen species in several tissues and organs (Oter et al. 2005, 2007) and thereby may accelerate cell and tissue damage. Finally, hyperbaric oxygen has been shown to increase the number of invasive inflammatory cells (Folz et al. 1999) and cause excessive production of reactive oxygen species in several tissues and organs (Narkowicz et al. 1993).

Oxygen treatments involving more than 40% oxygen have some adverse effects, for example, red blood cells are destroyed by active oxygen, and the binding of oxygen to hemoglobin in red blood cells decreases (Nagatomo et al. 2012a) although there are no data concerning the effects of oxygen concentration >40% on skeletal muscle properties. In contrast, mild hyperbaric oxygen at a low oxygen concentration, such as our value of 36%, does not increase oxidative stress (Ishihara et al. 2014). In addition, middle‐ear barotrauma, sinus pain, and painful joints, which are often induced by high pressure, do not happen under mild hyperbaric oxygen conditions. Mild hyperbaric oxygen also poses negligible risk of fire and explosion because of relatively low air pressure and oxygen concentration.

A previous study (Nagatomo et al. 2010b) suggests that mild hyperbaric oxygen (1.25 atmospheric pressure, 36% oxygen) is effective in reducing excessive levels of oxidative stress and C‐reactive protein in rats with type II collagen‐induced arthritis. Increased sympathetic activation in hypertensive rats is mediated by overproduction of highly reactive and toxic transient reactive oxygen species (Grassi 1998; Kishi et al. 2004). Mild hyperbaric oxygen reduces blood pressure and oxidative stress levels in spontaneously hypertensive rats (Nagatomo et al. 2010a), suggesting that mild hyperbaric oxygen decreases sympathetic activation in hypertensive rats. These results suggest that mild hyperbaric oxygen does not increase oxidative stress and instead inhibits elevated oxidative stress.

### Limitations of the study

In the present study, we measured mRNA levels, but not the protein levels, of *Pgc‐1α* in the soleus muscle. Thus, future studies will have to determine whether the protein levels were affected similarly. We can, however, definitively state that the increased mRNA levels of *Pgc‐1α* after a combination of pre‐ and postconditioning with mild hyperbaric oxygen was associated with the recovery of soleus muscle mass and oxidative capacity in hindlimb suspended rats.

## Conclusions

We conclude that a combination of pre‐ and postconditioning with mild hyperbaric oxygen can be effective against the atrophy and decreased oxidative capacity of skeletal muscles associated with hindlimb unloading.

## Conflict of Interest

None declared.
